# Effects of Acute Interval Exercise on Arterial Stiffness and Cardiovascular Autonomic Regulatory Responses: A Narrative Review of Potential Impacts of Aging

**DOI:** 10.3389/fcvm.2022.864173

**Published:** 2022-05-10

**Authors:** Wei-Long Liu, Yi-Yuan Lin, Toby Mündel, Chun-Chung Chou, Yi-Hung Liao

**Affiliations:** ^1^Department of Exercise and Health Science, National Taipei University of Nursing and Health Sciences, Taipei, Taiwan; ^2^School of Sport, Exercise and Nutrition, Massey University, Palmerston North, New Zealand; ^3^Physical Education Office, National Taipei University of Technology, Taipei, Taiwan

**Keywords:** high-intensity interval training (HIIT), intermittent exercise, arterial stiffness, pulse wave velocity, blood pressure, heart rate variability (HRV)

## Abstract

The physiological changes associated with aging deleteriously impact cardiovascular function and regulation and therefore increase the risk of developing cardiovascular disease. There is substantial evidence that changes in the autonomic nervous system and arterial stiffness play an important role in the development of cardiovascular disease during the aging process. Exercise is known to be effective in improving autonomic regulation and arterial vascular compliance, but differences in the type and intensity of exercise can have varying degrees of impact on vascular regulatory responses and autonomic function. There is still little evidence on whether there are differences in the response of exercise interventions to cardiovascular modulatory effects across the lifespan. In addition, acute interval exercise challenges can improve autonomic modulation, although the results of interval exercise on autonomic physiological parameters vary. Therefore, this narrative review focuses on evaluating the effects of acute interval exercise on blood pressure regulation and autonomic responses and also incorporates studies investigating different age groups to evaluate the effects of acute interval exercise on the autonomic nervous system. Herein we also summarize existing literature examining the acute cardiovascular responses to varied modes of interval exercise, as well as to further compare the benefits of interval exercise with other types of exercise on autonomic regulation and arterial stiffness. After reviewing the existing literature, it has been shown that with advancing age, changes in the autonomic nervous activity of interval exercise result in significant impacts on the cardiovascular system. We document that with advancing age, changes in the autonomic nerves lead to aging of the nervous system, thereby affecting the regulation of blood pressure. According to the limited literature, interval exercise is more effective in attenuating arterial stiffness than continuous exercise, but the difference in exercise benefits may depend on the training mode, intensity, duration of exercise, and the age of participants. Therefore, the benefits of interval exercise on autonomic and arterial stiffness improvement still warrant investigation, particularly the impact of age, in future research.

## Introduction

Recently, the National Health and Nutrition Examination Survey in the United States showed that approximately 70% of the elderly have hypertension (1). Cardiovascular disease risk increases progressively with age in elderly individuals (2, 3), and increased blood pressure usually accompanies the aging process (4–6). Blood pressure is associated with eutrophic remodeling, characterized by an increase in the wall-to-lumen ratio. Vasoconstriction, eutrophic remodeling with increased media-to-lumen ratio, changes in distensibility, decreased vasodilation reserve, and rarefaction characterizes small resistance arteries (7). With the development of the aging process, atrial stiffness leads to the hardening of the large blood vessel arteries in the body. The process is mainly characterized by structural changes in the arterial wall, including the development of fibrosis and degeneration of the elastin matrix (8, 9), and leads to a decrease in arterial elasticity. The decrease in the elasticity and the increase in the stiffness of blood vessels cause negative changes in cardiovascular function; previous epidemiological evidence suggests that cardiovascular risk increases after the age of 50 years (10). Moreover, central arterial stiffness is an important independent factor in cardiovascular risk among subjects of different populations (e.g., hypertensive, di§abetic, and >70 years of age) (11–14). Thus, although arterial stiffness was thought of as an inevitable consequence during normal aging, it is now considered to be a clinically relevant process. Implied that the prevalence of hypertension in the elderly will increase rapidly in the future, and the rate of cardiovascular disease (CVD) will increase accordingly (15). Therefore, developing preventive approaches to improve vascular health for different populations (including young and even older adults) should be considered.

Arterial stiffness is gradually being recognized as an important predictor and standard reference for hypertension, with aortic stiffness and arterial pulse wave reflection being determinants of elevated central systolic pressure, and arterial stiffness being strongly correlated with elevated blood pressure (16, 17). Pulse wave velocity (PWV) is an influential factor in the prediction of hypertension, and it is also a criterion for clinical assessment of arterial frontier stiffness (18). The acute and chronic effects of exercise intervention on PWV have been demonstrated. A previous study has observed a significant reduction in PWV 30 min after exercise in the acute post-exercise period, but the level of arterial stiffness returned to its basal level after 24 h (19). In addition, in a study of older individuals, a better reduction in PWV was observed after exercise (better fitness and more pronounced effect) than in the control group at 40% moderate intensity and 70% high-intensity mode, and this effect continued to be observed up to 90 min after exercise (20). The above findings thus suggest a single bout of exercise may acutely improve vascular function in young and elder populations. Moreover, a previous study has shown that endurance athletes show a significant increase in arterial stiffness after 3 months of detraining (21). Also, the decrease in arterial compliance caused by resistance exercise is observed to return to basal levels after 3 months of detraining (22). Furthermore, a decrease in carotid artery stiffness was observed after 8 weeks of swimming training in overweight adolescents, but this effect disappeared after 4 weeks of detraining (23). Age and blood pressure are the two main factors that affect PWV. Furthermore, there is strong evidence that the autonomic nervous system (ANS) has an essential role to affect CVD, and that aging and CVD are physiological factors that affect the regulation of pressure receptors in the ANS. When the ANS regulation is impaired, the cardiovascular system changes the arterial wall tension and increases blood pressure, leading to decreased arterial compliance and increased vascular sclerosis, resulting in the risk of CVD (24).

Given this, it is crucial to improve the management of hypertension to prevent future cardiovascular morbidity and mortality. A growing amount of research evidence suggests that physical activity interventions are effective and beneficial non-pharmacological treatments for patients with pre-hypertension and hypertension (25, 26). Prescribing exercise at moderate intensity may be more practical for most inactive adults with pre-hypertension or hypertension, and therefore most exercise prescription guidelines recommend a type of exercise that is classified as continuous moderate-intensity exercise (27, 28). Conversely, high-intensity interval training (HIIT) has not been comprehensively recommended by most clinical medical societies for the prevention or treatment of hypertension. Among the various types of exercise, interval exercise is an effective training method to improve cardiovascular health, and both interval and continuous exercise can reduce the risk of CVD. Even previous research has shown that interval exercise is more effective than continuous exercise in improving the risk of CVD (29). A more recent meta-analysis reported HIIT may be a more suitable alternative to traditional aerobic exercise to manage hypertension and the associated risk (30). However, it remains to be clarified whether different exercise prescriptions, such as training intensity and exercise duration, may have different effects.

Therefore, this narrative review is an accumulation of the existing literature on the assessment of the effects of acute interval exercise on blood pressure regulation and autonomic responses, and we also incorporate studies investigating different age populations to evaluate the effects of acute interval exercise on arterial compliance and the underlying physiological mechanisms for vascular function regulation. This review of the relevant literature provides a complete compilation of the current knowledge in the field. Here we address the following four main objectives through these following sequential topics, including (1) the effects of aging on cardiovascular regulation; (2) the effects of age differences on cardiovascular responses during acute interval exercise nomenclature; (3) the effects of acute intermittent exercise on autonomic regulation; (4) the comparisons of the acute effects of interval exercise and acute continuous exercise on arterial stiffness. Currently, several studies suggest that HIIT has not been fully recommended by most clinical societies for the prevention or treatment of hypertension. Among the various types of exercise, interval exercise is an effective training modality for improving cardiovascular health, and both interval and continuous exercise can reduce the risk of future cardiovascular disease, but there is no integrated review focusing on such issues. Therefore, we attempted to integrate the currently published evidence on the potential benefits of HIIT on arterial stiffness and the physiological mechanisms involved in modulating vascular function health in different age populations (e.g., young vs. old).

## Study Type and Literature Search Strategy

This review aims to summarize the effects of acute interval training on autonomic nerve response and pulse wave velocity. Studies were limited to those employed human participants and published in English-language, which were searchable in the electronic databases MEDLINE/PubMed, Web of Science (WOS), and Google Scholar conducted from inception to December 2021. The following keywords were used in the searches in conjunction with Medical Subject Heading (MeSH) terms, including autonomic nerve, heart rate variability, pulse wave velocity, and their related terms: acute, interval training, intermittent training, high intensity, sprint interval training, aerobic interval training, continuous training, moderate-intensity continuous exercise and HIIT. The following criteria had to be met to be included for detailed analyses, including a) the exercise intervention duration had to be a single-bout; b) directly compared acute interval training with continuous training; c) clearly defined outcome variables, particularly in autonomic nervous activity, heart rate variability (HRV), PWV as the primary outcomes. The exclusion criteria were as follows: a) articles using non-English-language sources; b) the original text was not obtained; c) statistical data could not be extracted; d) articles with insufficient information; e) documents with unreliable results; f) animal and cell researches.

## The Effects of Aging on Cardiovascular Regulation

Blood pressure is an essential component of the arterial load. When the heart contracts, the pressure generated by blood entering the aorta against the walls of the arteries is called systolic blood pressure (SBP) (31). Elevated blood pressure positively correlates with an increased risk of organ injuries, such as heart ventricle hypertrophy, coronary artery calcification, and increased carotid intima-media thickness. In the past, SBP >140 mmHg and diastolic blood pressure (DBP) >90 mmHg were defined as symptoms of hypertension, but recently the American Heart Association (AHA) has redefined the diagnostic criteria for hypertension so that SBP >130 mmHg and DBP >85 mmHg can be defined as hypertension, and epidemiological evidence also shows that hypertension is becoming younger (10, 31). Aging is a continuous process that affects the physiological decline of all the body's organ systems, thus increasing the incidence of degenerative diseases such as stroke, heart disease, and hypertension, which substantially increases the risk of death due to complications of related diseases (4). The development of hypertension due to the aging process is a major cause of cardiovascular disease and mortality (2, 3), and studies have shown that the control and treatment of hypertension can prevent cardiovascular disease in middle-aged adults (32).

The decrease in arterial wall elastin content and increase in arterial wall collagen during aging is accompanied by the development of arterial stiffness, which leads to an increase in SBP and a decrease in DBP (33). On the other hand, arterial wall elasticity is also influenced by the regulation of sympathetic nervous system activity, with reduced arterial wall elasticity contributing to hypertension and sustained increases in resting sympathetic activity leading to hypertension (34). Heart rate variability (HRV) is one of the important biomarkers used to assess ANS function, and evidence also suggests that a decrease in HRV is also a marker of aging and increased cardiovascular risk; some studies have shown a strong association between HRV and increased arterial stiffness (35). The ANS is an important regulator of the cardiovascular and metabolic systems, and aging is associated with changes in autonomic activity, leading to a decrease in baroreceptor reflex sensitivity (BRS) function, a decrease in parasympathetic activity in the sinus node, and an increase in sympathetic activity in the vascular system (36), as well as a decrease in BRS, which affects the environmental adaptation or visceral regulation of the ANS (37).

Blood pressure increases with aging, and hypertension is considered to be one of the degenerative diseases. Aging also causes an increase in visceral fat and blood leptin, and insulin resistance and excessive secretion of adiponectin impair endothelial cell function and cardiac function (5). The aging process has a negative functional and structural impact on the vessel, as reduced bioavailability of nitric oxide in the arterial endothelium, vasoconstriction, and increased secretion of endothelin will further increase SBP and mean arterial pressure (38). Aging is also associated with the development of chronic systemic inflammation and induces the secretion of inflammatory hormones, including tumor necrosis factor-alpha (TNF-α), monocyte chemoattractant protein 1 (MCP-1), induced- nitric oxide synthase, and interleukin-6 (IL-6), and also leads to leukocyte infiltration and the development of arterial stiffness (39, 40). Arterial stiffness and risk of cardiovascular disease increase with age, and increased arterial stiffness and peripheral vascular resistance in the central artery, increased vascular conduction velocity, and reduced arterial diastolic capacity can further contribute to abnormal arterial diastolic function. These changes are factors that cause cardiovascular disease (41). Studies have also shown that these physiological and biochemical factors affecting arterial vascular endothelial cell function accelerate the rate of cardiovascular aging, which leads to arterial stiffness and hypertension (5). On the other hand, when arterial compliance declines with advancing age in humans, the BRS is impaired; therefore, a reduction in BRS is a useful predictor of severe cardiovascular disease and cardiovascular mortality (42, 43). In macrocirculation, PWV is strongly associated with blood pressure, thus arterial stiffness can be assessed clinically by measuring pulse pressure velocity. In addition, studies have also shown that arterial stiffness can be used to prevent and predict the risk of vascular disease (44). The above describes the physiological mechanisms of aging on blood pressure, the regulation of autonomic nervous system function, and the pathological mechanisms of arterial stiffness.

## Effects of Age Differences on Cardiovascular Responses During Acute Interval Exercise Nomenclature

The carotid to femoral pulse wave velocity (cfPWV) is the gold standard for arterial stiffness measurements and is a reliable predictor of cardiovascular disease morbidity and mortality in the elderly. Aging and prolonged sedentary lifestyle can lead to an increase in PWV, and regular exercise training will help to reduce the risk of negative effects of sedentary lifestyle and aging on PWV (45). In a study by Shibata and Levine comparing sedentary elderly (65–77 years old), masters athletes (65–73 years old), and sedentary younger adults (20–42 years old), it was found that the “Modelflow aortic age” was higher in sedentary elderly than in sedentary younger adults. However, master athletes showed Modelflow aortic age younger than their chronological age (sedentary elderly had same age in Modelflow aortic age and chronological age) (46). These findings suggest that exercise training has a significant beneficial effect on improving age-related changes in aortic compliance, and also suggest that regular exercise habits may maintain the structural or functional disease of the aortic wall to mitigate the signs of aortic stiffness caused by aging (46). The restorative effect of exercise training on coronary artery and microvascular function may be attributed to improved endothelial cell function in the arteries, and older adults with exercise habits usually have relatively younger cardiovascular structure and function, which means that exercise training in older adults can reverse vasodilatory and microvascular dysfunction associated with aging and improve the blood flow response to acute exercise (47).

In a study recruiting middle-aged males (mean aged 48 years), the authors performed 45 min of moderate-to-high intensity interval exercise (intensity: 60–70% HRmax for 4 min and 90–100% HRmax for 1 min per set; completed 9 sets in total) and showed that moderate-to-high intensity interval exercise was effective in reducing systolic blood pressure and PWV in the lower extremities immediately after exercise (48). In another study, healthy young adults (mean age 27 years) were recruited to perform 30 min of moderate-to-high intensity intermittent walking exercise (intensity: 30% VO_2_max for 3 mins and 70% VO_2_max for 3 min per set; completed 5 sets in total), and changes in arterial stiffness after exercise were measured by analyzing cfPWV; the results of the above study revealed a reduction in cfPWV immediately after exercise (49). Through these two recent studies (48, 49), it appears that moderate-to-high intensity interval exercise exhibits comparable beneficial effects on cardiovascular compliance responses across young to middle-aged populations, particularly intermittent endurance exercise. Although based on the current body of literature, HIIT has been shown to have a positive effect on reducing arterial stiffness, not all studies have shown similar findings. In a recent study by Hortmann et al. (50), 15 young obese women (BMI=30–39.9 kg/m^2^) performed HIIT (85–95% of HRmax for 4 min and 65–75% of HRmax for 3 min, 4 sets) and continuous exercise (moderate-intensity continuous training, MCT) (65–75% of HRmax for 41 min). The results of this study showed that HIIT and MCT did not show a significant decrease in cfPWV after exercise (50). The above results suggest that HIIT appears to have no effect on post-exercise changes in cfPWV in obese women and further highlight that HIIT appears to show differential responses in different groups (48–50). Recently, a meta-analysis reported that HIIT was superior to MICT for improving night-time DBP and near-significant reductions in daytime SBP and DBP in favor of HIIT, indicating that HIIT may be a more potent therapy for improvements in blood pressure outcomes (30). However, there is no difference between either HIIT or MICT for changes to central arterial stiffness. Therefore, further studies are needed to determine the effect of different types of participants on arterial stiffness. Therefore, further studies are needed to determine the effect of different types of participants on arterial stiffness.

In particular, our review of the literature found that no studies have specifically examined the effects of HIIT interventions on changes in post-exercise arterial stiffness specifically targeting aging factors; therefore, it is still unclear whether HIIT has the same benefits for all age groups. To date, most of the relevant evidence examining the effects of exercise on arterial stiffness has been conducted in the same age group, and there is still a lack of research examining whether age differences in intermittent exercise-induced differences in cardiovascular function or arterial stiffness. Although many different types of HIIT patterns have been applied in research and routine exercise training, the duration, intensity, and work-to-rest ratios/intervals of these HIIT exercises vary widely. In addition, it is not yet fully understood which HIIT training modalities are effective in improving vascular flexibility and functional regulation, which will make it relatively difficult for exercise professionals to select appropriate HIIT exercise prescriptions for cardiovascular function.

## Effects of Acute Intermittent Exercise on Autonomic Regulation

The heartbeat is generated by the sinus node in the heart, which is regulated by the autonomic nervous system. The autonomic nervous system consists of sympathetic and parasympathetic nerves. When the sympathetic nerves are excited, the heart rate will increase to cope with the body's activities; when the parasympathetic nerves are excited, the heart rate will slow down, and the sympathetic and parasympathetic nervous systems will antagonize each other. Heart rate signals can be analyzed by special methods to assess the activity and regulation of the autonomic nervous system. There are two main types of such analysis methods: time-domain analysis, which is suitable for long time observations, and frequency domain analysis, which is suitable for short time analysis (51, 52). HRV is considered to be a safe non-invasive tool to monitor and assess the improvement in autonomic regulation to training adaptations, to be an important and stable indicator of autonomic nervous system function, and to measure resistance to stress. The ability of autonomic regulation and function can be quantified and assessed by simple and non-invasive HRV analysis (51, 52).

HRV decreases with age, and aging impairs the physiological structure of the autonomic and cardiovascular systems, leading to impairment of cardiac autonomic regulation during the aging process and a decrease in parasympathetic regulation of the autonomic nervous system, resulting in a decrease in vagal tone (53). Exercise training has protective and therapeutic effects on the autonomic nervous system, as skeletal muscle begins to contract during exercise, producing some vasodilatory metabolites (e.g., adenosine, prostaglandins, nitric oxide) that reduce the sympathetic-mediated vasoconstriction response (54). HRV analysis has been applied in many fields to reflect pre- and post-exercise autonomic conditioning and is one of the important methods to understand the physiological mechanisms of exercise performance and to assess autonomic conditioning (55).

HRV provides a clear assessment of the activity of many autonomic nerves. Radaelli et al. (56) studied 40 subjects with mild to moderate hypertension using HRV spectral analysis and found that hypertensive subjects had lower HF and higher LF than healthy subjects (56). Another study by Guzzetti et al. (57) also used HRV spectral analysis to compare the determination of the sympathovagal balance in subjects with and without hypertension, and the results were the same as in the previous study (57). The above-mentioned studies have shown that people with cardiovascular disease have higher sympathetic activity than normal people. On the other hand, short-term HRV analysis after acute exercise can effectively measure the role of the cardiac vagus nerve, and immediate post-exercise recovery is the best period to measure autonomic activity, and this allows the assessment of whether the autonomic nerve is abnormal for exercise (58). When the duration of exercise is shorter or the intensity is reduced, HRV can be rapidly restored to resting levels within minutes, but the longer the duration of exercise or the intensity is higher, the recovery of HRV can take up to several days to return to rest levels (59).

The low-to-high frequency ratio (LF/HF) can be used as a marker to assess cardiovascular autonomic balance, with an increase in LF/HF indicating a predominance of sympathetic balance and a decrease indicating a predominance of parasympathetic innervation (60). It has been established that higher exercise intensity is associated with increased changes in the autonomic balance after exercise. In a study by Parekh and Lee, the acute response in autonomic balance was compared between continuous exercise on a treadmill at 50% and 80% of VO_2_max, and the change in autonomic balance increased with higher exercise intensity, and the recovery time of vagal tone was longer. Conversely, the lower the intensity, the less the change in autonomic balance and the shorter the recovery time of vagal neural regulation (61).

Exercise training causes changes in HRV, and several studies have been conducted to compare the effects of acute interval exercise and continuous exercise on autonomic balance. Several studies have shown that interval exercise has a more positive effect on the acute cardiovascular response than continuous exercise. However, the results of studies comparing interval exercise and continuous exercise on post-exercise effects on autonomic balance are divergent. Andrade et al. compared the cardiovascular and HRV effects of endurance exercise (20 min at 80% of maximum heart rate) and high-intensity interval exercise (1 min of exercise at 90% of maximum heart rate and 1 min at 60% of maximum heart rate, repeated 10 times). Results showed that both the HIIT trial and the endurance trial caused significant decreases in TP, LF, and HF; however, LF/HF showed a significant increase only in the HIIT trial, but no difference in the endurance group (62). In the study by Schaun and Del, 12 adult males performed 8 sets of HIIT exercises with 10 s of exercise + 10 s of rest intervals on a bicycle ergometer at an intensity of 70% Pmax (maximal oxygen consumption relative to power). The results of the study showed that the HRV indices (RRmean, RMSSD, HR, and LF) for both time and frequency domain analysis decreased immediately after exercise; however, the LF/HF did not decrease immediately, but only 24 h after exercise. Since HIIT involves very high-intensity exercise, the reason for this change may be that high-intensity exercise increases sympathetic stimulation and depresses the parasympathetic vagus nerve. Therefore, Schaun and Del's finding that LF/HF decreases only 24 h after exercise should be a “supercompensation” phenomenon of vagal regulation (63). However, compared to the previous two studies, Cerda-Kohler, Pullin and Cancino-López et al. held a different opinion (64). In their study, participants were asked to perform 8 km of continuous endurance exercise and interval exercise on a treadmill respectively, even though the interval exercise pattern used in this study was more intense (100% of the peak treadmill speed) than continuous endurance exercise (63% of the peak treadmill speed). Both exercise regimens differed in intensity but were equal in volume (distance run); the results of this study showed significant differences in LF/HF before and after exercise between the two regimens in terms of acute response (64). Taken together, we speculate that the inconsistent findings of exercise-induced HRV changes reported in different studies may be attributed to differences in subjects' mean heart rate, physical fitness, physique, or basal state of autonomic activity; since these factors are also important factors influencing basal HRV, the results of exercise response to HRV also vary.

Most studies to date have found (Table 1) that participants can experience a decrease in autonomic activity after either acute interval exercise or continuous endurance exercise. However, interval exercise appears to reduce sympathetic and parasympathetic activity more substantially than continuous endurance exercise (62). Additionally, it was found that single bouts of high-intensity exercise improved HRV modulation. Therefore, the difference in the degree of HRV changes induced after a single exercise session may depend on the training status, exercise intensity, and exercise duration of the participants; however, there are no definite results on the effect of altered autonomic recovery status after exercise (59, 65).

**Table 1:** Comparison of high-intensity interval exercise and continuous exercise on autonomic nervous system to acute exercise.

**References**	**Participant characteristics**	**Experimental group (case)**	**Exercise intervention**	**Outcome indicators**	**Outcomes (Δ%)**
Kaikkonen et al. (59)	Male athletes, *N* = 8 age:26 yrs	(IV) two interval interventions two (CO) continuous interventions	(IV85) 7 x 3 min at 85% VO_2_max with 2-min recovery intervals (IV93) 7 x 3 min at 93% VO_2_max with 2-min recovery intervals (CO80) 21 min at 80%VO_2_max (CO85) 21 min at 85%VO_2_max	HF LF TP	The changes of HRV recovery percentage between MPE and the 30-min recovery following both types of exercise. (IV85) HF ↑2%, LF ↑9%, TP↑2% (IV93) HF ↑1%, LF ↑14%, TP↑2% (CO80) HF ↑1%, LF ↑6%, TP↑2% (CO85) HF ↑1%, LF ↑17%, TP↑2%
Stuckey et al. (65)	Healthy male, *N* = 10 age:19 yrs	(1W) single sprint intervals (4W) multiple sprint intervals	(1W) one Wingate, 1W (4W) four Wingate interspersed with 4 min of light cycling, 4W	HF LF	The changes of HRV recovery percentage between MPE and the 60-min recovery following both types of exercise. (1W) HF↓32%, LF ↑27% (4W) HF↓31%, LF ↑35%
Cerda-Kohler et al. (64)	Healthy male, *N* = 7 age:33 yrs	(INTT) intermittent exercise (CONT) continuous exercise	(INTT) an intermittent exercise for 8 km at 100% of the peak treadmill velocity (30 min of 15 s running, interspersed with 15 s of passive rest). (CONT) a continuous exercise for 8 km at 110% of the lactate threshold.	LF/HF	(INTT) MPE: LF/HF ↑ (INTT) post 24 h: LF/HF ↔ (CONT) MPE: LF/HF ↑ (CONT) post 24 h: LF/HF ↔
Andrade et al. (62)	Runners (4 male and 4 female), *N* = 8 age: 23 yrs	(HIIT) high-intensity interval training (EE) endurance exercise	(HIIT) 1 min of exercise at 90% maximal heart rate per 1 min of rest, 10 times (EE) 20 min at 80% maximal heart rate	TP LF HF LF/HF SBP/DBP	(HIIT) MPE: TP↓77%, LF↓71%, HF↓87%, LF/HF ↑220%, SBP↑13%, DBP ↔ (EE) MPE: TP↓37%, LF↓34%, HF↓41%, LF/HF↔, SBP↑12%, DBP ↔

*VO_2_max, maximal oxygen consumption; LF, low frequency; HF, high frequency; TP, total power; LF/HF, a ratio of low frequency to high frequency; MPE, immediately post exercise; SBP, systolic blood pressure; DBP, diastolic blood pressure; ↑, increase; ↓, decrease; ↔, no change*.

## Comparisons of the Acute Effects of Interval Exercise and Acute Continuous Exercise on Arterial Stiffness

Arterial stiffness is a major contributor to the development of cardiovascular disease, and endurance exercise has been shown to reduce arterial stiffness (48, 66). However, differences in training parameters such as frequency, intensity, duration, and type of exercise intervention can have different degrees of effect on arterial stiffness, and the types of endurance exercise are mainly divided into continuous and interval endurance exercises. Therefore, it is important to understand the differences in the effects of continuous or interval exercise on arterial stiffness to help professionals better select which type of exercise to use as a reference treatment tool (67).

Several studies have shown that endurance exercise can improve vascular health by reducing arterial stiffness, central and peripheral blood pressure, and improving arterial compliance (48, 66, 68). Many studies have reported that single, moderate, and high-intensity aerobic exercise can improve arterial stiffness. In a previous study, Kingwell et al. (68) performed a single 30-min cycling session at 65% of maximal oxygen intake and showed an increase in aortic blood flow and carotid pressure and a decrease in aortic conduction velocity, suggesting that acute continuous endurance exercise can reduce PWV and thus increase systemic arterial compliance. This suggests that acute continuous endurance exercise can reduce PWV and thereby increase systemic systemic arterial compliance; this effect may be related to vasodilatory mechanisms (68). Systemic arterial compliance = Ad/[R(Ps- Pd)], where Ad is the area under the blood pressure diastolic decay curve (from end-systole to end-diastole), R is total peripheral resistance, Ps is the end-systolic blood pressure, and Pd is the end-diastolic blood pressure. In another moderate-intensity study, continuous (30 min at 50% of VO_2_max) and intermittent walking exercises (intensity: 30% VO_2_max for 3 min and 70% VO_2_max for 3 min, 5 sets) were performed in 14 healthy adolescents, and it was found that the carotid pulse conduction velocity decreased by 21% in the intermittent walking group and by 14% in the continuous walking group after exercise; this indicates that intermittent exercise is more effective than continuous exercise in reducing systemic arterial stiffness (49). However, in another study using high-intensity exercise, Rakobowchuk et al. (69) recruited nine healthy men who underwent a single Wingate test and an interval exercise test consisting of four Wingate tests. However, the effect of interval exercise consisting of four Wingate sessions on arterial stiffness was not significantly more effective than one Wingate session; the authors speculated that this may be because the Wingate test is a high-intensity exercise, and the maximum effect on vascular elasticity was already achieved during the first Wingate session, so the fatigue level had already accumulated to a certain level in the following sessions, and the exercise level was not able to perform at the best level (69). Together, both interval and continuous endurance exercise can have significant improvements in arterial stiffness; however, the benefits of high-intensity interval exercise on arterial stiffness are not higher than those of a single session of high-intensity exercise (e.g., Wingate sprint exercise).

On the other hand, in addition to single, moderate- and high-intensity exercises that can reduce arterial stiffness, low-intensity aerobic exercise also has a significant effect on the reduction of arterial stiffness (48, 69). In a previous study by Wang et al. (66), a single 30-min session of continuous and interval cycling (2x15 mins with 20min of rest) at 35% of HRR at the same intensity was performed on 15 healthy young adults, and it was found that the cardio-ankle vascular index (cAVI) in the interval exercise group decreased by 16% immediately after exercise and remained low after 40 min, whereas the continuous exercise group decreased by 15% and returned to baseline cAVI after 40 min (69). This suggests that low-intensity exercise can also improve arterial stiffness, and it also suggests that there is a significant difference between interval and continuous exercise even if the exercise intensity is the same, but the effect on arterial stiffness after exercise and the time effect may be different (69). This suggests that low-intensity exercise can also improve arterial stiffness, and that the effect of exercise on the decrease in arterial stiffness after exercise is influenced by the type of exercise even if the exercise intensity is the same for interval and continuous exercise (69). Similar results were found in another study where subjects performed interval and continuous endurance cycling exercises at the same time at moderate intensity. The interval exercise group showed a 16% decrease in lower extremity PWV immediately after exercise, while the continuous exercise group showed a 7% decrease, and the decrease after the interval exercise group was greater than the continuous exercise group. The authors concluded that the exercise patterns of IE and CE, with greater changes in blood flow after IE than CE, resulted in higher fluctuations in cardiac output during IE, which led to greater pulse wave reflection and release of nitric oxide, resulting in a better effect of IE than CE (48).

Regarding the possible effects of long-term HIIT exercise training on vascular function, a recent review and analysis of the relevant literature showed that long-term HIIT exercise training interventions were more effective in promoting vascular function than moderate-intensity continuous exercise, with long duration intervals (≥2 min) appearing to be more effective (70). Furthermore, HIIT has also been reported to be more effective in reducing the augmentation index (AIx), a reduction in AIx represents due to wave reflection, thereby reducing arterial stiffness and vasodilatation, eventually, myocardial burden. Therefore, HIIT can effectively reduce AIx, which also indicates recurring periods of reduced wave reflection may cumulatively result in cardiovascular benefits (71). It is worth noting that a recent study on the changes in vascular function after HITT training in elderly people found that compared to moderate-intensity continuous exercise training, HITT did not improve the aortic wave conduction rate (cfPWV) (72), but significantly improved cardiopulmonary fitness, ventricular output ratio (ejection fraction), and insulin sensitivity (73).

Altogether, interval exercise was more effective than continuous exercise in reducing systemic arterial stiffness, but the degree of effect may vary depending on the subject, exercise activity, intensity, and duration of the intervention on the acute effects of arterial stiffness, and therefore the issue remains to be investigated in subsequent studies. However, when comparing interval and continuous exercise, the exercise benefits of interval exercise appear to be more pronounced (Table 2).

**Table 2:** Comparison of high-intensity interval training and continuous training on arterial stiffness in response to acute exercise.

**References**	**Participant characteristics**	**Experimental group (case)**	**Exercise intervention**	**Outcome indicators**	**Outcomes (Δ%)**
Rakobowchuk et al. (69)	Healthy male, *N* = 7 age: 20 yrs	(SW) single Wingate (MW) multiple Wingate	(SW) 1 Wingate for 2h at 40W (MW) 4 Wingate for 2.5 h at 40W	cPWV PWVLL	(SW) MPE: cPWV ↑75%, RTB: 20 min (SW) MPE: PWVLL ↓ 21%, RTB: 45 min (MW) MPE: cPWV ↑ 62%, RTB: 20 min (MW) MPE: PLLWV ↓ 29%, RTB: 45 min
Wang et al. (66)	Healthy young male, *N* = 15 age: 21 yrs	(CON) non-exercise control (IE) interval exercise trial (CE) continuous exercise	(CON) no exercise intervention (IE) two 15-min of cycling within 20-min rest (CE) continuously 30-min of cycling at 35% HRR	CAVI	(CON) MPE: CAVI ↔ (CON) post 40 min CAVI ↔ (IE) MPE: CAVI ↓16% (IE) post 40 min CAVI and did not RTB (CE) MPE: CAVI ↓15% (CE) post 40 min within RTB
Perissiou et al. (74)	Older adults, *N* = 51 age:72 yrs	low-fit mid-fit high-fit	(CON) underwent control (MOC) moderate-intensity continuous cycling at 40% PPO (HIIT) 12 × 60 s bouts at 70% PPO, with each interval separated by 60 s at 10% PPO	PWV RM	RM high-fit MOC vs. HIIT ↓5% PWV high-fit HIIT vs. CON ↓5% high-fit MOC vs. CON ↓3%
Okamoto et al. (49)	Healthy young male, *N* = 14 age: 27 yrs	(IW) interval walking (CW) continuous walking	(IW) 5 sets of 3-min walks at 30% and 3-min walks at 70% of VO_2_max (CW) a continuous exercise for 30 min at 50% VO_2_max.	cfPWV	(IW) MPE: cfPWV↓21%, RTB: 60 min (CW) MPE: cfPWV↓14%, RTB: 30 min
Peres et al. (48)	Healthy male, *N* = 15 age:48 yrs	(IE) interval cycling exercise (CE) Constant exercise	(IE) 9 sets of 4-min cycling at 60–70% of HRmax and 1-min cycling at 90–100%, for 45 min (CE) a continuous exercise for 45 min at 50% VO_2_max.	cPWV PWVUL PWVLL BP	(IE) MPE: cPWV↓6.5%, PWVUL↓8%, PWVLL↓16%, BP↓8% (CE) MPE: cPWV↑2%, PWVUL↑3%, PWVLL↓7%, BP↓2%
Magalhães et al. (75)	Patients with type 2 diabetes, *N* = 80 age=59 yrs	(CON) non-exercise group (HIIT) combined high-intensity interval training with RT (MCT) combined moderate continuous training with RT	(CON) underwent control (HIIT) 17 HIIT 1-min bouts at 90% of the VO_2_reserve and 1 set of 10–12 repetitions of upper and lower limbs exercises, 3 times/week, for 1 years. (MCT) continuous cycling for 34 min at 40 % - 60% of the HRR and 1 set of 10–12 repetitions of upper and lower limbs exercises, 3 times/week, for 1 years.	CD PWV CR PWV CIMT	(HIIT) CIMT↓1.1%, CR PWV↓10.0%, CD PWV↓11.2% (MCT) CIMT↓1.4%
Hortmann et al. (50)	Young obese women, *N*=15 BMI=30 39.9 kg /m^2^	(CON) control condition (HIIT) high-intensity interval training (MCT) moderate-intensity continuous training	(CON) 30 min at rest in the sitting position (HIIT) 4 × 4 min at 85%−95% of HRmax, interspersed with t3 min active recovery periods at 65%-75% of HRmax (MCT) 41 min at 65–75% of HRmax	cSBP cDBP cfPWV	(CON) MPE: cSBP ↔, cDBP ↔, cfPWV↔ (CON) post 60 min: cSBP ↔, cDBP ↔, cfPWV↔ (HIIT) MPE: cSBP ↔, cDBP ↔, cfPWV↔ (HIIT) post 60 min: cSBP ↓5%, cDBP ↔, cfPWV↔ (MCT) MPE: cSBP ↓5%, cDBP ↔, cfPWV↔ (MCT) post 60 min: SBP ↓5%, cDBP ↔, cfPWV↔

*cPWV, central PWV; PWVLL, pulse wave velocity in lower limb; PWVUL, pulse wave velocity in upper limb; MPE, immediately post exercise; RTB, returned to baseline; HRR, Heart Rate Reserve; CAVI, cardio-ankle vascular index; PWV, pulse wave velocity; PPO, peak power output; RM, reflection magnitude; cfPWV, carotid-femoral pulse wave velocity; VO_2_max, maximal oxygen consumption; HRmax, maximum heart rate; RT, resistance training; CD PWV, carotid distal pulse wave velocity; CR PWV, carotid radial pulse wave velocity; CMIT, intima-media thickness; cSBP, central systolic blood pressure; cDBP, central diastolic blood pressure; ↑, increase; ↓, decrease; ↔, no change*.

## Concluding Remarks and Future Directions

**Figure 1:**
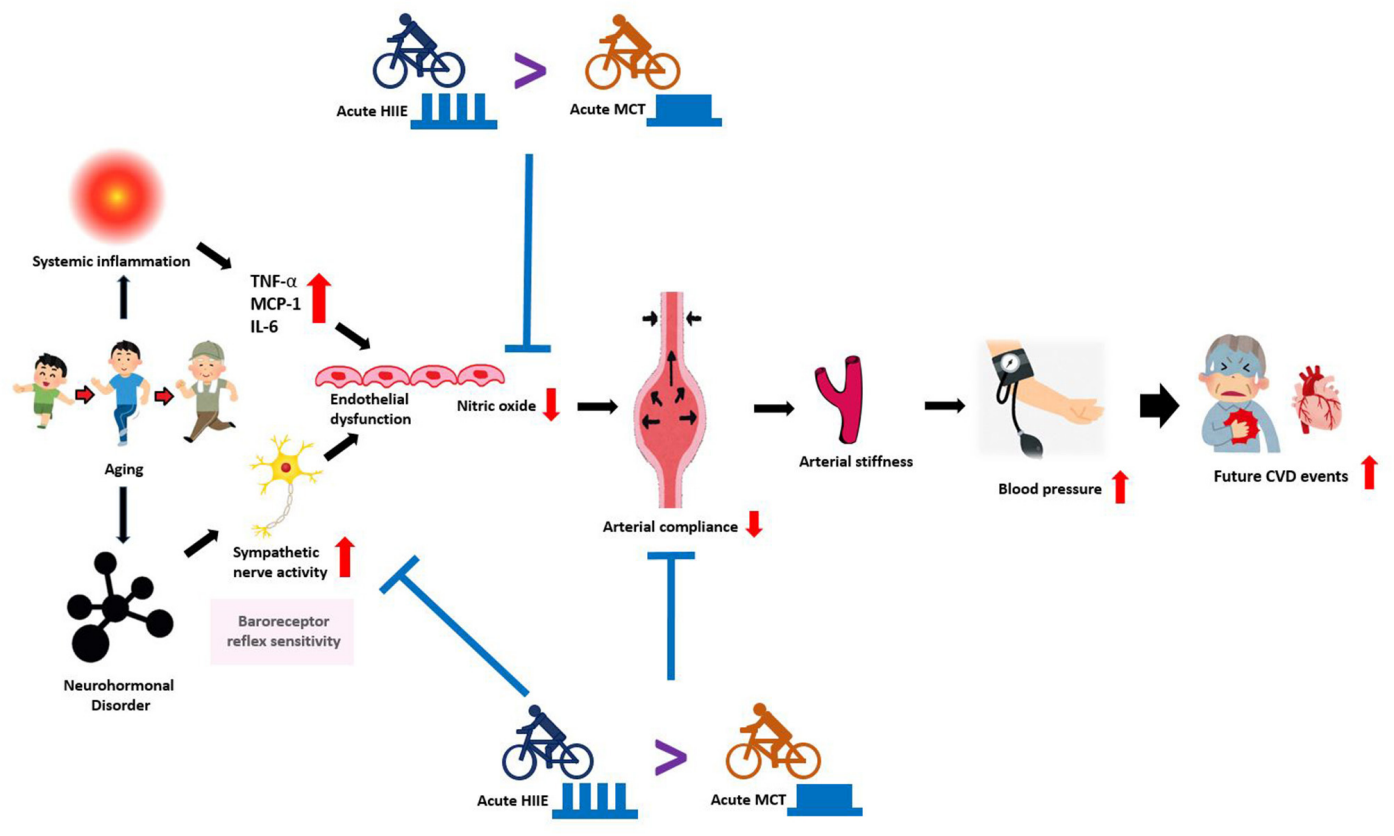
An overview of the mechanisms mediating age-related vascular dysfunction and how HIIT and MCT can improve vascular function by decreasing sympathetic nerve activity. With advancing age, several adverse changes occur in the arterial system that drives the development of CVD. Aging is associated with reduced baroreceptor reflex sensitivity (sympathetic nervous activity) and endothelial dysfunction, marked by reduced nitric oxide and increased inflammatory mediators (TNF-α, MCP-1, IL-6, etc.). Together, these processes induce vascular dysfunction, featuring: neurohormonal disorder, reduced arterial compliance, increased arterial stiffness that drives the development of high blood pressure, which also increases the risk for future cardiovascular events. In agreement with scientific evidence, both HIIT and MCT training improves vascular dysfunction by reducing sympathetic nervous activity and increasing nitric oxide. In contrast, HIIT is more efficacious for improving vascular function and increasing baroreceptor reflex sensitivity than MCT. HIIT: high-intensity interval training; MCT: moderate continuous training CVD: cardiovascular disease; TNF-α: tumor necrosis factor-α; MCP-1: monocyte chemoattractant protein-1; IL-6: interleukin-6.

Physiological changes caused by aging have many negative impacts on cardiovascular regulations. Although the results of the reviewed studies revealed that the studied exercise modes are varied in terms of intensity, interval pattern, and type, interval exercise and continuous exercise are both capable of acutely improving HRV regulation and reducing PWV. Figure 1 illustrates and summarizes an overview of the physiological mechanisms mediating age-related vascular dysfunction and how HIIT and MCT can improve vascular regulatory function by decreasing sympathetic nervous activity. Although studies have reported varying degrees of responses to cardiovascular-related physiological markers in response to acute interval exercise, interval exercise appears to be comparable or even more effective in improving cardiovascular health than other types of exercise. In other words, the strength-duration curve matters, to the skeletal muscle of the limbs, the smooth muscle of the blood vessels, and the cardiac muscle of the heart, together with the autonomic control of heart rate. In this narrative review, the main focus is to compile the acute effects of HIIT and MCT after exercise. However, based on a review of the available research evidence, it is unclear how long the immediate effects of these two different forms of exercise on vascular compliance modulation can be sustained, and therefore more recent studies are needed in the future. Declines in exercise performance and cardiovascular adaptability and its physiological determinants are inevitable with aging. Furthermore, a recent meta-analysis reported that HIIT or SIT induced more significant VO_2_max gains than MICT, suggesting that interval training is comprehensive evidence for a viable training strategy to improve cardiorespiratory fitness in healthy middle-aged and older adults (76).

On the other hand, it is noteworthy that master athletes are mostly routinely trained and regularly participate in sports competitions, and this group also shows superior physical fitness, cardiovascular adaptations, and superior athletic performance compared to their age-matched peers (77, 78). Given this, master athletes should be categorized as a special group. However, it remains unclear whether there are differences in the benefits and mechanisms of vascular compliance modulation in master athletes after acute HIIT and MCT exercise. In addition, does HIIT produce different acute changes in vascular compliance in masters athletes compared to untrained individuals of the same age? These research questions also suggest that future studies could be conducted to further investigate.

Most of the existing studies have examined the effects of interval exercise on HRV or PWV in participants within similar age ranges or in specific populations (e.g. obesity, chronic diseases, etc.,). However, it has to be noted that there are only a few studies that consider the effect of age differences on these physiological variables in response to acute interval exercise, so the effects of aging on interval exercise-induced acute cardiovascular changes are still unclear. Future studies are warranted to further investigate the acute response of interval exercise on autonomic regulation and arterial stiffness among groups of different ages to fully explore the underlying mechanisms for impacts of aging on autonomic regulation and arterial stiffness to acute interval exercise.

## Author Contributions

W-LL, Y-YL, TM, C-CC, and Y-HL contributed to the conceptualization and edited and revised the manuscript. W-LL and Y-YL contributed to the methodology. C-CC and Y-HL contributed to the collection, synthesis, and interpretation of the data. W-LL, Y-YL, C-CC, and Y-HL drafted the manuscript. All authors approved the final version of the manuscript.

## Funding

This research was partially supported by the Ministry of Science and Technology (MOST, Taiwan; Y-YL: 110-2410-H-227-001; C-CC: 110-2410-H-027-019; Y-HL: 109-2628-H-227-002-MY3). The APC was partially funded by the Ministry of Science and Technology (MOST) and the National Taipei University of Nursing and Health Sciences (NTUNHS).

## Conflict of Interest

The authors declare that the research was conducted in the absence of any commercial or financial relationships that could be construed as a potential conflict of interest.

## Publisher's Note

All claims expressed in this article are solely those of the authors and do not necessarily represent those of their affiliated organizations, or those of the publisher, the editors and the reviewers. Any product that may be evaluated in this article, or claim that may be made by its manufacturer, is not guaranteed or endorsed by the publisher.
